# Intraoperative torsion control with radiological cortical thickness parameters in distal tibial shaft fractures: a cadaveric study

**DOI:** 10.1007/s00402-025-06178-z

**Published:** 2026-02-02

**Authors:** Lena Keppler, Richard Zaccaria, Christian Zeckey, Konstantin Küßner, Eduardo M. Suero, Carl Neuerburg, Maximilian Weigert, Wolfgang Böcker, Alexander M. Keppler

**Affiliations:** 1 Musculoskeletal University Center Munich (MUM), LMU University, Munich, Germany; 2 RoMed Hospital, Rosenheim, Germany; 3 StaBLab, LMU Munich, Munich, Germany

**Keywords:** Distal tibial shaft fractures, Intramedullary nailing, Tibial malrotation, Intraoperative torsional assessment, Cortical step sign, Diameter difference sign, Fluoroscopy, Cadaveric study

## Abstract

**Aim:**

Intramedullary nailing is a common and safe procedure in the treatment of distal tibial shaft fractures. It is often accompanied with maltorsion of the tibia due to insufficient intraoperatively available and objective diagnostic tools. Therefore there´s high need for reliable tools for intraoperative torsional control. Radiographic parameters such as the Cortical Step Sign (CSS) and the Diameter Difference Sign (DSS) may serve for diagnosing relevant maltorsion intraoperatively.

The aim of this study was to investigate the effect of maltorsion on CSS and DSS parameters in a distal tibial fracture model and to construct a prognostic model to detect maltorsion.

**Methods:**

A distal tibial shaft fracture (AO/OTA type “A”) was set on 19 human tibias. Torsion was gradually adjusted from 0° to 30° in external and internal torsion. Images were acquired with a C-arm in two planes and transferred to a PC for measurement of medial cortical thickness (MCT), lateral cortical thickness (LCT), tibial diameter (TD) in a.p., and anterior cortical thickness (ACT), posterior cortical thickness (PCT), and transverse diameter (TD lat.) in lateral view of the proximal and distal fragment.

**Results:**

For the various levels of torsion significant differences for each of the values of the examined variables could be shown. Highest visibility was found for ACT, TD a.p. and TD lat. Highest correlation of radiographic difference and maltorsion was found in internal torsion (TD lat./ TD a.p.). A threshold of less than 2 mm led to a probability to detect maltorsion of 0.7. ROC for the lateral model was better than for the a.p. model (0.866 vs. 0.829). TD lat. performed best regarding ROC in single parameter evaluation (0.778). Best prediction for relevant maltorsion was obtained with TD a.p. and TD lat.

**Conclusion:**

CSS and DSS are useful tools for detection of maltorsion in distal tibial shaft fractures. The parameters can be easily collected and therefore represent promising parameters for intraoperative torsional control.

## Introduction

Distal tibial shaft fractures account for approximately 15–20% of all tibial fractures and represent one of the most challenging fracture entities due to their metaphyseal anatomy, limited soft-tissue envelope, and high risk of maltorsion (axial, rotational, and angular) [1, 2]. Several epidemiological studies have shown an increasing incidence, particularly in high-energy trauma and sports injuries [3, 4]. Accurate fracture reduction is the main prerequisite for recovery and fracture healing. Minimally invasive techniques using intramedullary nails for closed reduction and fracture fixation are an established method for treating tibial shaft fractures. It´s the most popular and widely used procedure as it minimizes extensive soft tissue dissection and possibly allows earlier weight-bearing compared to open reduction and plate osteosynthesis [5, 6].

However, intraoperative evaluation of fluoroscopic-guided fracture reduction after closed reduction with intramedullary nails is often difficult. Therefore, a common complication after intramedullary nailing of tibial fractures, despite the use of fluoroscopy, is postoperative maltorsion of the fracture site. Several large studies looking at tibial intramedullary (IM) nails showed a high risk of postoperative maltorsion ranging from 22 to 55% [7–9]. In most studies tibial maltorsion is defined as a torsional difference of 15° or more between the proximal and distal main fragment.

Consequences include, in addition to cosmetic issues, significant limitations of gait and stability function, and alteration of joint biomechanics with the development of osteoarthritis in the knee and ankle [5, 10, 11].

For this reason, tibial maltorsion is a common indication for revision surgery after tibial fractures. Although postoperative low-dose CT scans have become the gold standard for the detection of torsional errors, torsional control remains challenging using intraoperative diagnostic tools. Studies on how to avoid maltorsion are scarce [12, 13]. The need to establish intraoperative tools for torsional control during intramedullary-nailing is greatly felt [14]. The cortical step sign (CSS) and the diameter difference sign (DDS) are established radiographic parameters used to assess torsional alignment during intramedullary fixation of long bone fractures [15]. Originally developed for femoral shaft fractures and later validated in proximal and midshaft tibial regions, they offer simple, reliable intraoperative markers of torsional deviation. While the CSS and the DDS have been previously validated in proximal and midshaft tibial fractures, no data exist for distal tibial shaft fractures [16, 17]. Additionally, clinical reports reinforce the consequences of unrecognized distal tibial maltorsion. A notable case report by Takase et al. described symptomatic tibial maltorsion after intramedullary fixation requiring corrective osteotomy, highlighting the persistent need for reliable intraoperative torsional control methods [18]. Therefore, a dedicated assessment of CSS and DDS in distal tibial shaft fractures is essential to establish region-specific thresholds and improve intraoperative detection of clinically significant maltorsion.

The present cadaveric study aims to address this unmet need by quantifying CSS and DDS parameters in distal tibial shaft fractures at varying degrees of torsion and by developing a predictive model for clinically relevant maltorsion in this anatomically distinct region. We hypothesize that the distinct anatomical characteristics of the distal tibia, its progressively narrower and more triangular-to-oval cross-section, thinner cortices, and reduced cortical asymmetry lead to measurably different radiographic behavior of CSS and DDS compared with the mid-diaphysis and proximal tibia. Therefore, threshold values and diagnostic accuracy derived from proximal or midshaft studies cannot be directly applied to the distal tibia, necessitating region-specific evaluation.

## Methods

In order to simulate a distal tibial shaft fracture (AO/OTA type 42-A3c), a transverse osteotomy was performed at the junction of the medial to the distal thirds for nineteen fresh frozen human cadaveric tibia specimens. After the osteotomy a nail (ETN^®^ Tibial Nail 9 × 315 mm, DePuy Synthes, Umkirch, Germany) was inserted for intramedullary fixation. By using a protractor the torsional difference of the two fragments was gradually set at 5°, 10°, 15°, 20°, 25° and 30° of external and internal torsion. Therefore, we fixed the proximal part of the fracture and inserted two K-wires (diameter 2.0 mm) strictly parallel at the front edge of the distal Tibia serving as static indicators to mark the direction of the planned correction. No manipulation was applied to the wires at any time. The correct torsion was checked by two independent measurements using a protractor.

**Fig. 1:**
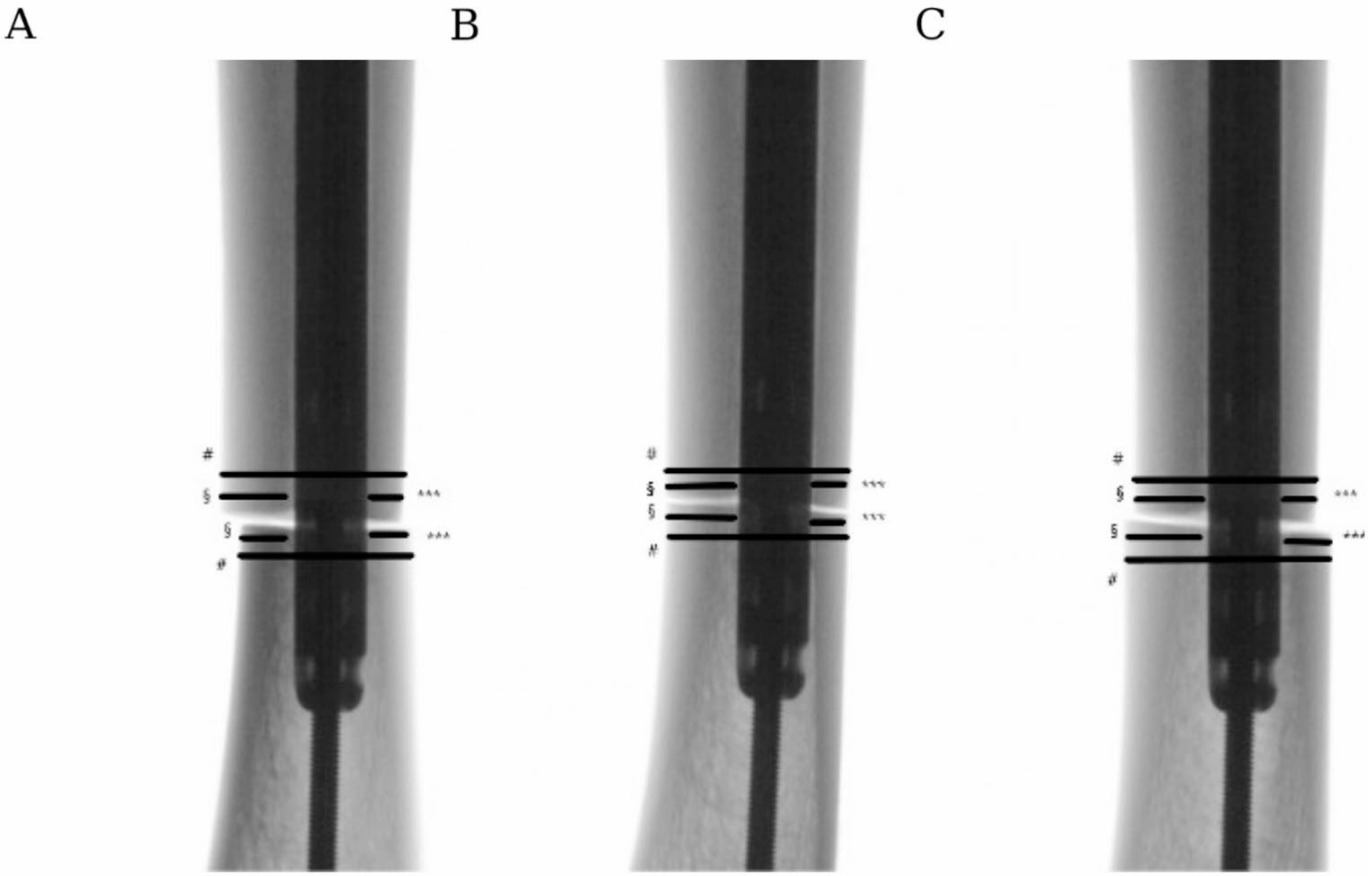
View of the different torsion in AP view. **A** 20° external torsion, **B** 0° (neutral position), **C** 20° internal torsion. #= Tibial shaft diameter (TD); *= Lateral cortical thickness (LCT), **= Medial cortical thickness (MCT)

The K-wires served as pointers. By using a mobile C-arm (Ziehm RF3D, Ziehm Imaging, Nuremberg, Germany) 2D radiographic images were acquired in true anteroposterior (AP) and lateral views of the osteotomy site as referenced by the posterior tibial crest. For measurements, the generated X-ray images were then processed using RadiAnt^®^ DICOM Viewer (Medixant. RadiAnt DICOM Viewer [Software]. Version 5.5.0. November 2019. URL: https://www.radiantviewer.com). The known diameter of the inserted nail served as reference for the calibration. The CSS was evaluated measuring the following parameters: the medial cortical thickness (MCT), lateral cortical thickness (LCT), anterior cortical thickness (ACT) and posterior cortical thickness (PCT) of the tibia proximal and distal to the transverse osteotomy in a true anteroposterior (AP) and lateral view (Fig. 1). As already shown in prior studies a cortical thickness difference of 0.6 mm between the proximal and distal bone segments was defined as a positive CSS [19].

Furthermore, for the analysis of the DDS, the transverse diameter of the femoral bone segment proximal and distal to the induced osteotomy was measured in true AP and lateral view (Tibial diameter [TD]). Analogous to CSS a difference of 0.6 mm between the proximal and distal femoral diameter was considered as a positive DDS.

The nineteen fresh frozen human cadaveric tibia specimens (4 female and 15 male donors) were harvested by the Institute of Forensic Medicine of the Ludwig-Maximilians-University of Munich. The median age of the donors was of 61.84 years (SD ± 14.4 years) and the median body mass index of 27.7 kg/m2 (SD ± 4,0.5 kg/m2). Table 1 shows the diameter of each specimen at the osteotomy site.

The study was approved by the local Ethics Committee of the Medical Faculty (Nr. 18–184) and all procedures were followed in accordance with relevant guidelines.

**Table 1:**
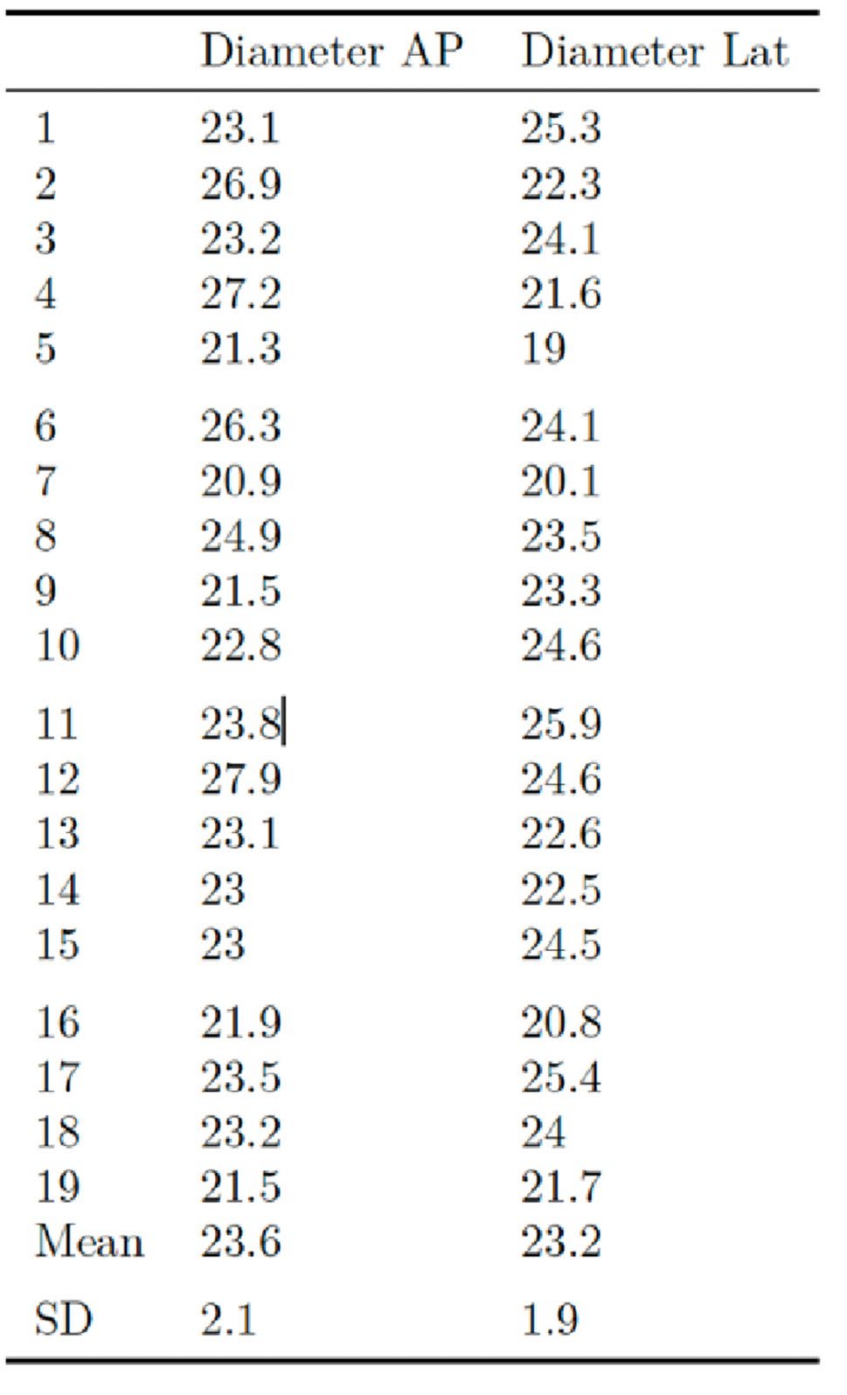
Diameter of each tibia specimen at the osteotomy site in the anteroposterior (AP) and lateral radiographic views

## Results

### Measurements of radiographic parameters

In the evaluation of the a.p. and lateral X-ray images in external torsion, significant differences were found for each of the parameters obtained (*p* < 0.001). In a.p. view it was found that visibility at clinically relevant 15° was significantly higher for TD (89.47%) and MCT (63.16%) than for LCT (52.63%). MCT, LCT and TD increased with further external torsion in a.p. (Table 2). The lateral view of the external torsion in 15° showed a very high visibility for ACT (89.47%) (Table 3). PCT and TD lat. performed worse. ACT and TD increased with further external torsion in the lateral view.

**Table 2:**
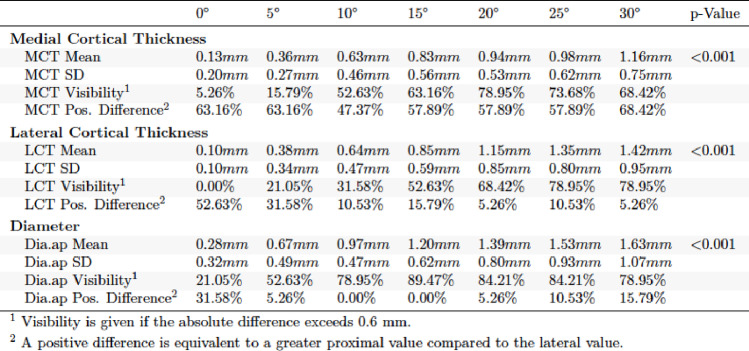
Absolute differences in external torsion a.p. view

**Table 3:**
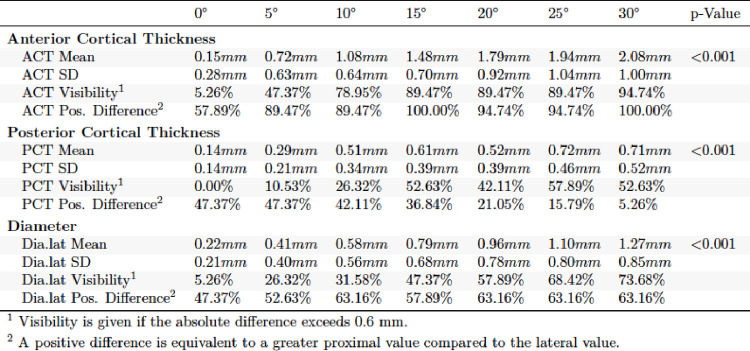
Absolute differences in external torsion lateral view

For internal torsion in a.p. and lateral view highly significant differences were observed (*p* < 0.001). MCT (52.63%) and TD (73.68%) showed higher visibility than LCT (31.58%) in the a.p. view (Table 4). In the lateral view TD performed better than ACT and PCT regarding visibility (Table 5).

**Table 4:**
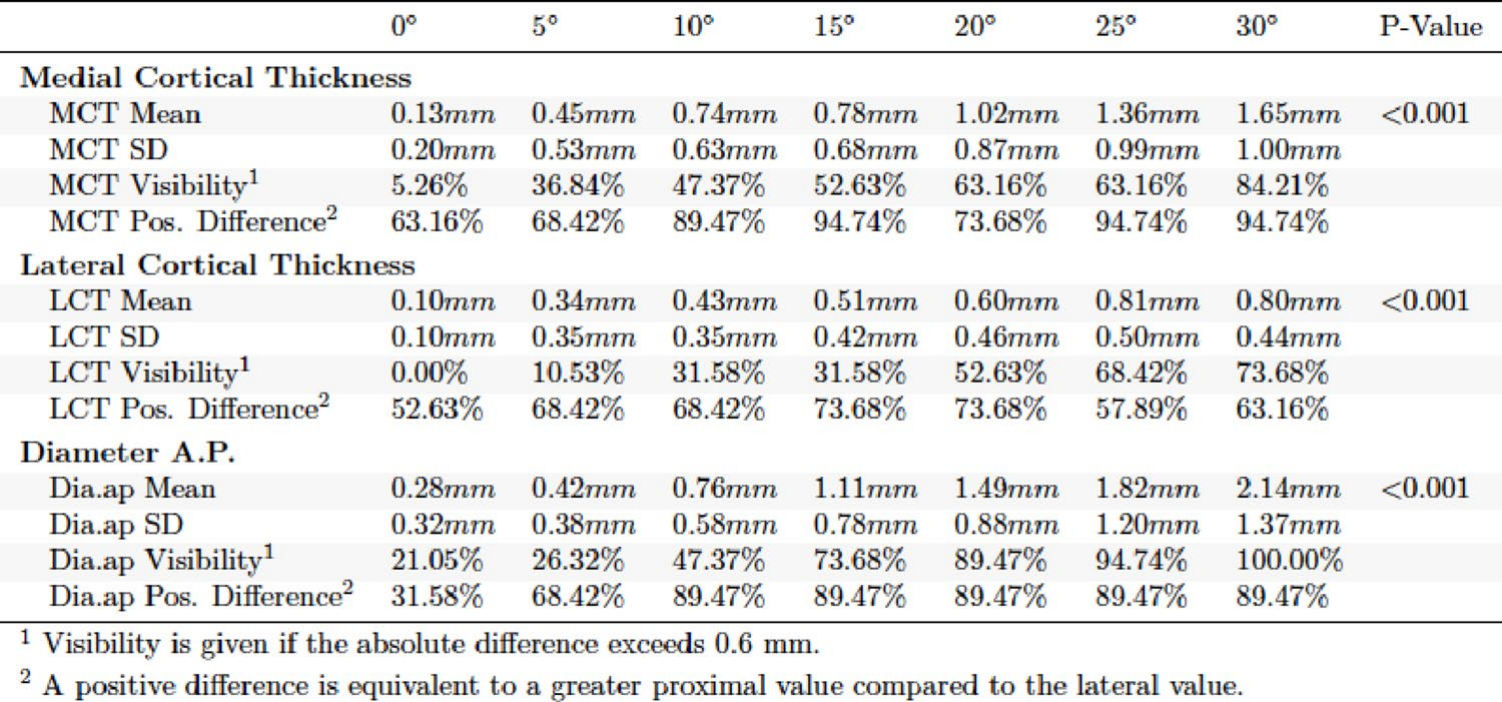
Absolute differences in internal torsion a.p. view

**Table 5:**
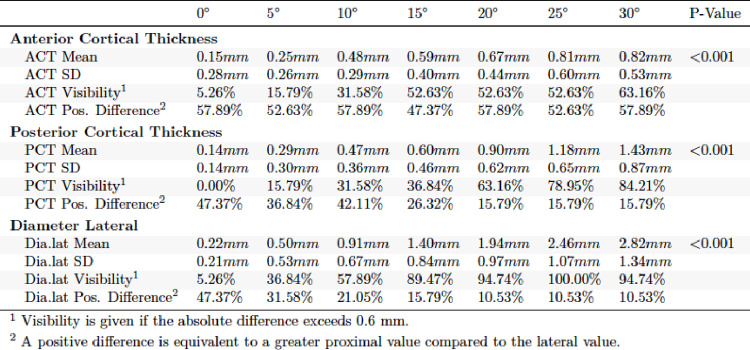
Absolute differences in internal torsion lateral view

**Fig. 2:**
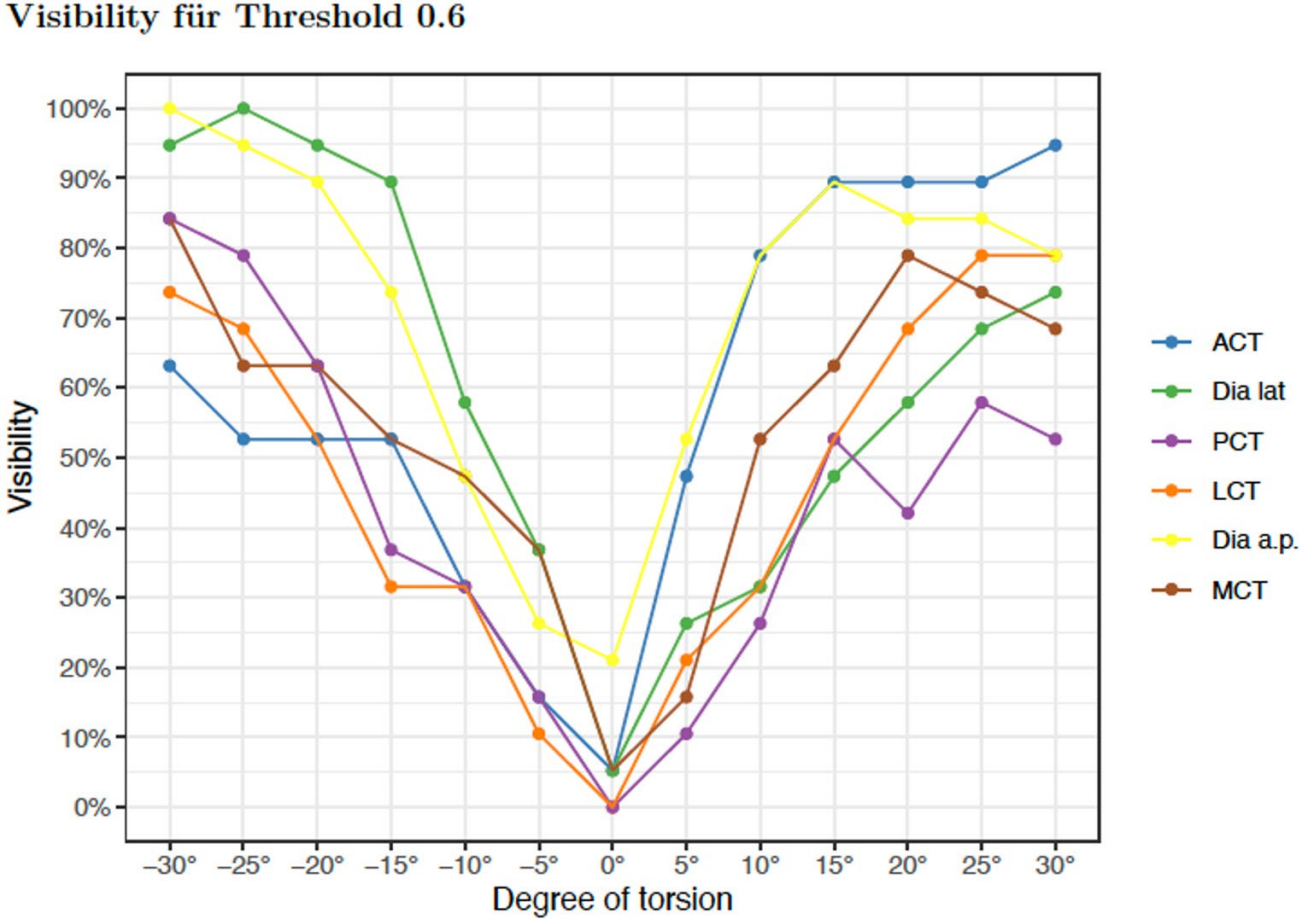
Visibility of torsion for threshold 0.6 mm

Positive difference was highest in ACT in external torsion lateral view (100%) and lowest in TD external torsion, a.p. view (0%). A positive difference indicates that the proximal fragment measures larger in the lateral view compared to the anteroposterior (a.p.) view. To better examine visibility at threshold 0.6 mm Fig. 2 was established.

The negative values on the X-axis in Fig. 2 indicate internal torsion, while the positive values describe external torsion. There it can be shown that the collected parameters perform best at maximum internal and external torsion (30° each). For the clinically relevant 15°, the highest values for visibility in external torsion are found for ACT and TD a.p., and for TD lat. and TD a.p. in internal torsion.

### Correlation between radiographic parameters and tibial maltorsion

In external torsion correlation of absolute differences showed the highest values for TD lat. (0.56, 0.54), TD a.p. (0.55), and ACT (0.5). In internal torsion highest values were found for TD lat. (0.79; 0.75) and TD a.p. (0.68).

**Fig. 3:**
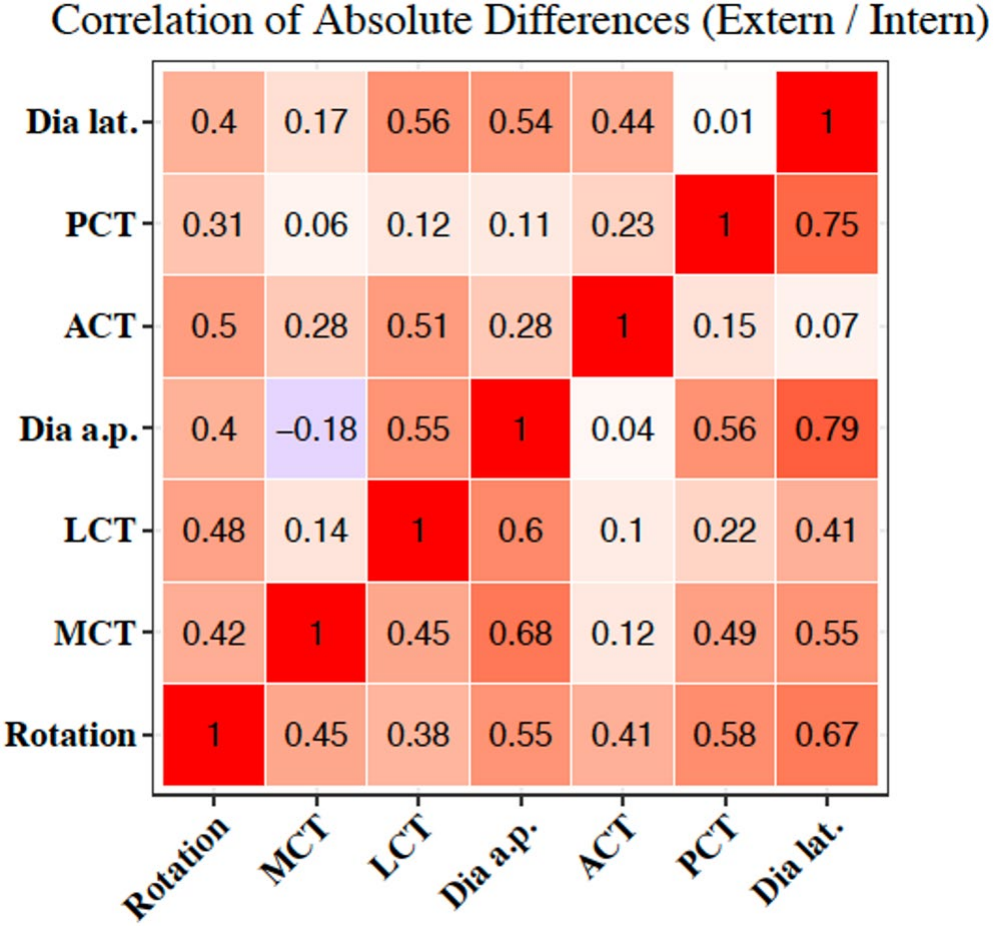
Correlation of absolute radiographic differences and maltorsion. Upper triangle: External torsion. Lower triangle: Internal torsion

In this context, TD lat. is the best parameter for assessing torsion. Highest correlation was shown for external torsion by TD lat./LCT and for internal torsion by TD lat./TD a.p., followed by TD lat./PCT (Fig. 3).

### Probability of maltorsion by measurement threshold

Logistic regression models were used to find threshold values for the different torsion parameters collected. Based on the results for the clinically relevant 15°, PCT performs better than LCT, and MCT in terms of threshold. Here, low values can be shown in which a torsion deviation becomes visible and clinically relevant. To achieve a probability of 0.8 in each case, 1.44 mm for PCT, 1.94 mm for MCT, and 1.57 mm for LCT were observed. Specificity is 0.97 or greater for the three parameters.

In the a.p. view 2.26 mm (TD) were necessary to reach a probability of 0.8 (specifity 0.98). PPV was 0.86. ACT and TD lat. performed similar. Please see Table 6 for the results.

**Table 6:**
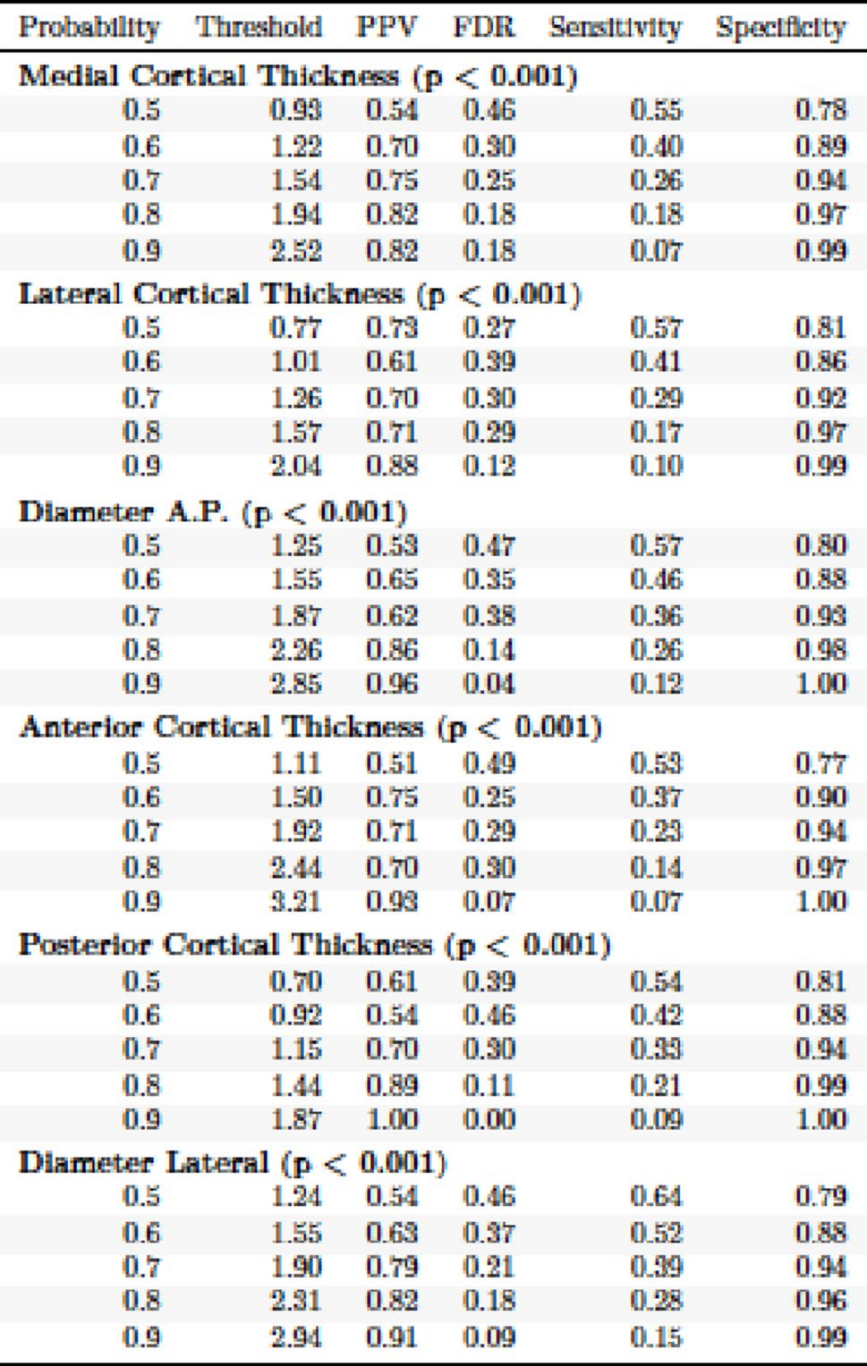
Probability of tibial maltorsion at each threshold value for each of the analyzed parameters

### ROC analysis of multiple logistic regression models

**Fig. 4:**
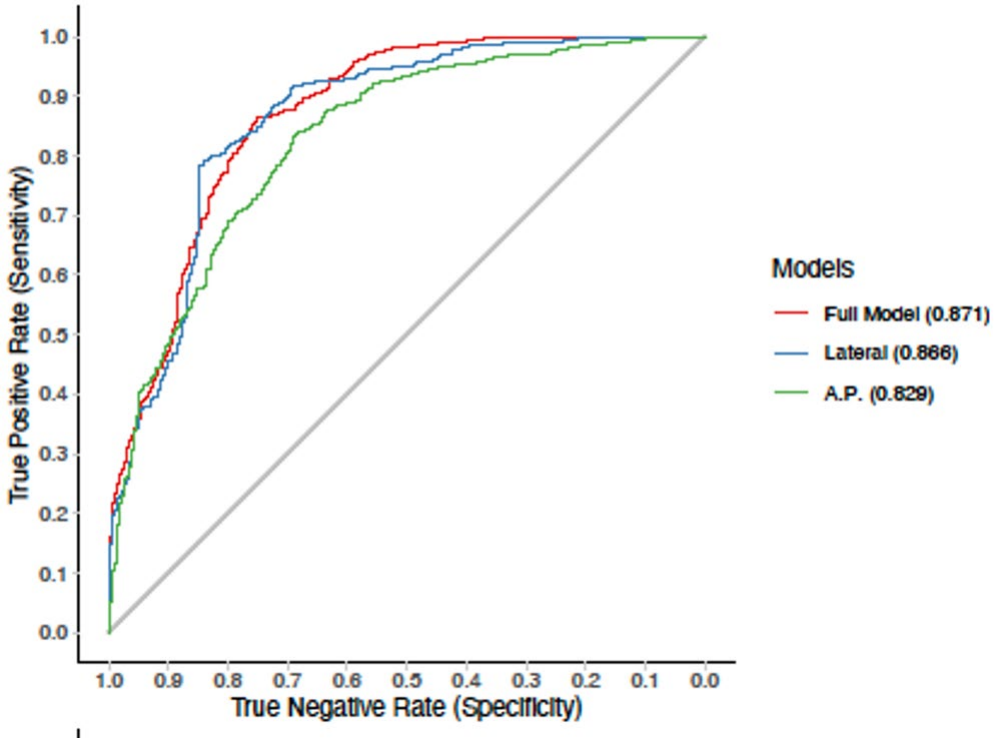
Comparison of receiver operating characteristic (ROC) curves of the univariate models of tibial maltorsion as a function of radiographic cortical and diameter parameters

In addition to the results already presented, ROC curves were generated to better analyse the thresholds and their influence on sensitivity and specificity. High AUC values were obtained for both the a.p. and lateral models (0.866 vs. 0.829). When combining the two models, the AUC can be increased to 0.871. Therefore, the combination of the two models has an even higher predictive power than the single models. However, the lateral model performs slightly better than the a.p. model when considered individually (Fig. 4).

**Fig. 5:**
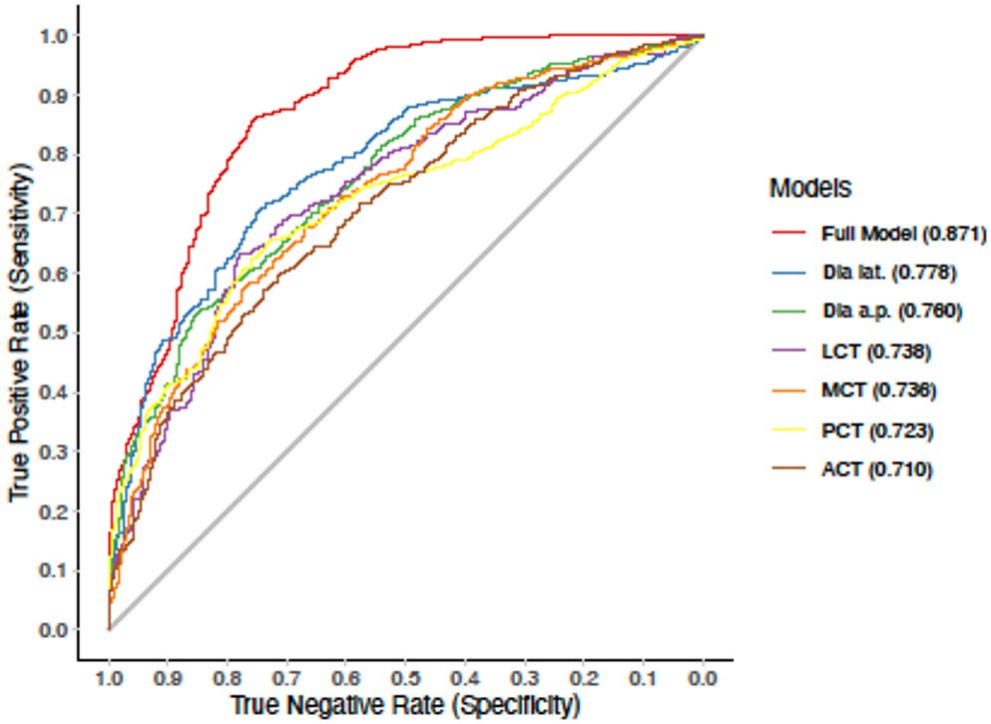
Receiver operating characteristic (ROC) curves of the models of tibial maltorsion as a function of the combination of variables in the anterior posterior or lateral views demonstrating improved sensitivity and specificity

A closer look at the ROC of the individual parameters surveyed reveals that TD lat. and TD a.p. perform better than the other values. The AUC for TD lat. is 0.778 and 0.76 for TD a.p. The combination of the parameters (full model) raises the AUC to 0.871. Please see Fig. 5 for more details.

## Discussion

To the best of our knowledge this is the first study quantifying and describing the usability of the Cortical Step Sign and Diameter Difference Sign to detect maltorsion in the distal tibial shaft. The presented study is part of a series of studies regarding probability of CSS and DDS after intramedullary nailing in tibial fractures of various heights. The studies for proximal and tibial midshaft fractures have already been published and showed high probability and feasibility of the examined parameters [16, 17]. In the presented study the results provided the first reproducible data on the validity of the Cortical Step Sign and Diameter Difference Sign in distal tibial shaft fractures.

We demonstrated the visibility and probability for maltorsion in different parameters in external and internal torsion. External torsion showed higher visibility whilst internal torsion showed higher values for correlation. Threshold values for obtaining a probability of 0.8 were low (< 2 mm) in certain parameters. Regarding ROC the lateral model showed higher AUC.

Tibial maltorsion is one of the more common pitfalls in intramedullary nailing of the tibia and occurs in up to 30% [14]. Torsional control is challenging because tibial fractures are often accompanied by soft tissue injury and swelling or because of various positions of the leg on the operating table. In addition, closed reduction procedures and the presence of multiple fracture fragments can complicate appropriate alignment and interpretation of fluoroscopic images. It is important that clinicians consider these factors and consider alternative methods of assessing alignment to ensure appropriate treatment as maltorsion of the tibia leads not only to revision surgery, but also to secondary osteoarthritis, non-union, and significant functional limitations [10, 20, 21]. Regarding these factors there´s high need for preventing maltorsion.

Although there is no clearly defined limit for the tolerance of torsion errors in the current literature to date, in many studies deviations of more than 10°−15° are, however, considered an indication for revision surgery [7, 22, 23]. Therefore, in the study presented, a limit value of 15° was established.

In the current literature few attempts and algorithms can be found to prevent or detect maltorsion [24, 25]. For example Inci et al. and Takase et al. described two different options for intraoperative torsional control with e.g. the help of an external target device or the insertion of two K-wires [18, 26]. Another approach was published by Bleeker et al. in 2022 [27]. There, a standardized intraoperative fluoroscopy protocol named the ‘C-arm Rotational View (CARV)’ of both legs is used. This protocol has been tested in cadavers so far. The authors assume that the CARV-protocol has proven accurate and reproducible results resulting in reduced maltorsion. Although radiation exposure was between 0.001 and 0.003 mSv, X-ray is needed on both legs which raises radiation exposure and surgery time. Furthermore, this protocol only works in patients with almost symmetric tibias leading to symmetric landmarks and can therefore be a limitating factor [28].

In contrast to tibial maltorsion, studies have been published on successful intraoperative detection for maltorsion in femoral shaft fractures, yet [15, 29, 30]. In the postoperative setting, CT imaging continues to be considered the gold standard. However, this is associated with increased radiation exposure, as well as revision surgery. The goal must therefore be to detect torsional errors intraoperatively and with simple and appropriate means.

With the Cortical Step Sign and the Diameter Difference Sign, which firstly were described by Krettek et al. in 1998 for femoral shaft fractures, it is now possible to find relevant indications for the presence of maltorsion intraoperatively with simple means, namely two-plane X-ray fluoroscopy of the injured leg, and to correct this during the operation [12].

These parameters have already been used successfully in cadaveric femoral, and now in proximal as well as in tibial mid-shaft fractures. Our study continues these experiences and results obtained, and shows that the CSS and DDS can also be applied in distal tibial shaft fractures. Across our series of studies evaluating CSS and DDS along the tibia, clear regional differences in radiographic behavior have emerged. In contrast to the proximal and mid-shaft tibia where pronounced cortical asymmetry and larger diameters provide robust fluoroscopic landmarks the distal tibia presents a fundamentally different anatomical environment. Its narrower profile, more rounded or triangular cross-section, and thinner cortices reduce the magnitude of cortical step changes and diameter differences under torsional stress. These structural distinctions explain why threshold values, parameter visibility, and correlation strength differ from those observed in our previously published proximal and mid-shaft models.

Our findings confirm that intraoperative torsion assessment cannot be generalized across the tibial shaft. The distal region requires its own set of validated measurements due to its unique morphology and higher susceptibility to malrotation. By comparing the present results with earlier models, a consistent anatomical gradient becomes apparent: the more proximal the fracture, the more pronounced the cortical steps and diameter changes whereas the distal tibia demonstrates smaller but still diagnostically usable values. This gradation underscores the necessity of region-specific data rather than anatomical extrapolation.

The clinical relevance of these distinctions is underscored by published reports of symptomatic distal tibial maltorsion after intramedullary fixation. The case report by Takase et al. illustrates how even moderate maltorsion can lead to functional impairment requiring revision surgery, emphasizing the need for reliable intraoperative tools [18]. Our study provides such data for the distal tibia for the first time, complementing established evidence from the proximal and midshaft regions.

Taken together, this study completes a comprehensive anatomical analysis of CSS and DDS along the tibial shaft and demonstrates that these radiographic parameters are feasible and clinically applicable tools even in the morphologically challenging distal tibia. The region-specific thresholds identified here may support surgeons in preventing maltorsion and reducing the need for corrective procedures.

There are some limitations to our present study. In this study we only examined one osteotomy height and fracture type in the distal tibial shaft. In this case further studies with different fracture types and height would be useful to prove our results. However, our model offers clear advantages. Due to the standardized osteotomy and the subsequent equal fracture height and fracture type, the results can be compared very well. Furthermore, our model proves that the procedure to quantify tibial maltorsion is relatively easy to perform and requires only a small amount of technical equipment.

Our model already showed very good, and reliable results in proximal and tibial mid-shaft fractures. In the already published studies the probability of the examined parameters and their clinical relevance was proven.

We’d like to highlight that our results show low threshold values indicating a high probability of clinically relevant maltorsion in contrast to proximal and mid-shaft fractures. Therefore, especially in distal tibial shaft fractures anatomical reposition seems to be mandatory.

We hypothesize that the distinct anatomical configuration of the distal tibia—characterized by a narrower, more triangular cross-section and thinner cortices—directly affects the magnitude and detectability of CSS and DDS parameters. This anatomical variability provides a plausible explanation for the differences observed when compared with our proximal and mid-shaft studies. To support this thesis, further studies need to be done on this subject.

## Conclusion

We have examined the Cortical Step Sign and Diameter Difference Sign at various levels of tibial maltorsion after intramedullary nailing in the distal tibial shaft. In addition, we have constructed a model to accurately predict tibial maltorsion based on intraoperative two-plane radiographic parameters. Comparison of our three published studies to date on the tibia prove that the CSS and DDS can be used along the tibial shaft to detect maltorsion. The results further suggest that specific anatomical characteristics of the tibia may influence these findings. In particular, the tibial shaft becomes increasingly oval in its distal portion, which alters the cortical contours visible on fluoroscopy. As a consequence, the CSS may need to be interpreted differently at various tibial levels, and slight variations in its appearance should be considered a normal reflection of the bone’s changing cross-sectional geometry. However, the CSS and the DDS show feasibility and applicability even in the distal tibial shaft and can therefore be promising tools for detecting maltorsion in tibial fractures *intraoperatively*.

With these tools the incidence of clinically relevant maltorsion and the need for additional revision procedures could be significantly decreased.

## Data Availability

No datasets were generated or analysed during the current study.
